# Diet Data Collected Using 48-h Dietary Recall: Within—and Between-Person Variation

**DOI:** 10.3389/fnut.2021.667031

**Published:** 2021-07-06

**Authors:** Sinara Laurini Rossato, Sandra Costa Fuchs

**Affiliations:** ^1^Postgraduate Programs in Epidemiology, School of Medicine, Universidade Federal Do Rio Grande Do Sul, Porto Alegre, Brazil; ^2^Department of Nutrition, Harvard T. H. Chan School of Public Health, Boston, MA, United States; ^3^Graduation Course of Collective Health, Institute of Geography, Universidade Federal de Uberlândia, Uberlândia, Brazil; ^4^PREVER National Institute of Science and Technology, Hospital de Clínicas de Porto Alegre, Porto Alegre, Brazil

**Keywords:** nutrient intake variation, 48-h dietary recall, nutrition method, day-to-day nutrient variation, within-person variation, between-person variation

## Abstract

**Background and Aims:** Forty-eight-hour dietary recall is a valuable source of information regarding food consumption in a population-based sample. This method covers 2 consecutive days in a single interview. Nevertheless, the number of assessments and the sample size necessary to estimate usual intake are unknown. We aimed to assess sources of variation, sample sizes, and numbers of days necessary to estimate usual nutrient intake using the 48-h dietary recall.

**Methods:** This was a population-based cross-sectional study including 237 participants, 11–90 years old, selected using multistage probabilistic sampling to obtain data using 48-h dietary recall. Analysis of variance was used to calculate within- and between-person variation and determine the statistical parameters necessary to calculate sample size and the number of days required to calculate the usual energy and nutrient intake.

**Results:** Within-person variation was generally lower than between-person variation, except for calcium (${\text{CV}}_{\text{w}}^{2}$ = 40.8; ${\text{CV}}_{\text{b}}^{2}$ = 38.4%), magnesium (${\text{CV}}_{\text{w}}^{2}$ = 27.4; ${\text{CV}}_{\text{b}}^{2}$ = 18.7%), and monounsaturated fat (${\text{CV}}_{\text{w}}^{2}$ = 20.0; ${\text{CV}}_{\text{b}}^{2}$ = 17.3%) for the entire group and magnesium for women (${\text{CV}}_{\text{w}}^{2}$ = 28.3; ${\text{CV}}_{\text{b}}^{2}$ = 91.8%). The number of days and sample size required to determine usual energy and nutrient intake varied substantially with gender and age (e.g., vitamin C in women *N* = 9, in men *N* = 1,641).

**Conclusions:** Energy and nutrient intake assessment using the 48-h dietary recall misrepresents within-person variation but can generate acceptable results for between-person variation. The calculation of sample size and number of days required to determine usual energy and nutrient intake might have been affected by inadequate assessment of the within-person variation.

## Introduction

Food records and 24-h dietary recall methods provide detailed information regarding food and nutrient intake; they help determine dietary intake in population settings (1–3). They also examine sources of bias and random variation in collecting nutrient intake data (1). Several studies assessed the within- and between-person variation through the food record and 24-h dietary recall and demonstrated the need to collect dietary information for at least 2 days (1–7) to capture the within-person variation (1, 6). A follow-up study showed within-person variation in energy intake throughout the year, including a weekly residual variance of 2–26% of energy intake and a systematic variation of energy intake according to the days of the week, coincident with a slight variation in between-person variation (4). Other studies revealed different sources of variation per population settings (5–8), including the variation among days, weeks, or seasons (9) and well-marked differences per season in Brazil (5) that depended on sex and age (7).

Random variation is commonly observed in short-term methods. This variation affects statistical analysis and hypothesis testing because it increases the data dispersion, although the nutrient or food intake average does not change (9, 10). One day of dietary intake assessment does not represent usual food and nutrient intake (10, 11); therefore, collecting more than one repeated measure has become common practice in diet surveys to capture day-to-day variation, also known as random variation of food intake, which is usually large (11). The greater the number of days of dietary intake assessment, the closer the data dispersion approaches the variation of usual dietary intake (10–17). After several 24-h dietary recalls, the variation becomes more stable (10–17). Still, the number of repetitions required depends on the population in the study, gender, culture, diversity of food intake, and the level of accuracy expected.

Palaniappan et al. showed that the energy variance ratio was much lower among men (0.49) than women (1.76) (16). Oh and Hong reported that older adults required more dietary records in Korea than in western countries, resulting in culture-dependent eating habits. The authors concluded that the number of repetitions required for nutrients depended on the type of nutrients, gender, and the level of accuracy expected. For instance, to assess energy intake with 5% accuracy, it would be necessary to record 97 days of dietary assessments among men and 128 among women; however, for riboflavin, 240 days would be necessary for men and 455 for women. The number of days required to assess the usual nutrient intake was smaller if lower accuracy is accepted. For example, in the same study, energy intake among men could be captured with 1 day of assessment if 50% accuracy was deemed acceptable (17).

This method of evidence collection has guided study design, sample size calculation, and strategies for data collection to reduce bias and random variation across populations (4–18). It has emphasized collecting dietary data in non-consecutive days to capture the within-person variation for food or nutrient intake (10). Nevertheless, data collection using these methods is practically infeasible in large or prospective studies because of the number of replications required to ensure their accuracy, resulting in expensive data collection and complexity of the analysis to manage random variation and bias (4–9). Alternatively, 48-h dietary recall enables collecting 2-day dietary data in a single individual assessment, increasing the response rate and expanding the within-person variation. The paucity of studies using the 48-h dietary recall allied with specific characteristics of this methodology implies that its ability to capture within-person variation is not known.

Nevertheless, a few studies have already adopted 48-h dietary recall. A recent study comparing the derivation of food patterns using various methods for data collection of diet showed that 48-h dietary recall appeared to be superior to single 24-h dietary recall because it allowed the generation of food patterns with a reduced number of interviews (18). Compared to a Food Frequency Questionnaire, the 48-h dietary recall was found to estimate the protein intake accurately (19). Rehm et al. found that 48-h dietary recall was superior to a single food record (20).

Regardless of whether a 48-h dietary recall is a valid method for capturing the natural variation of food and nutrient intake in population settings, the method could gather information of 2 days (24-h dietary recall) in one interview, optimizing time and increasing response rate. Therefore, in the present study, we evaluated the sources of variation, estimating the sample size and number of days required to detect the usual intake of energy, macronutrients, cholesterol, total fiber, and micronutrients by gender, age, seasons, and periods of the week in data collected using the 48-h dietary recall method.

## Materials and Methods

This was an exploratory analysis using data collected to validate a semi-quantitative Food Frequency Questionnaire in southern Brazil. Dietary intake was assessed using 48-h dietary recall to better capture the within-person variability in a single evaluation. This analysis hypothesized that a 48-h dietary recall would capture within-person variation better than a single 24-h dietary recall, but not as much as two non-consecutive 24-h dietary recalls. Because secondary data were used in this study, nutrient intake calculated with the 48-h dietary recall used in this data collection was compared with 2 days of 24-h food recalls according to the available literature. In this manner, our analysis aimed to support decision-making for future studies based on the strengths and limitations of 48-h dietary recall.

### Participants

**Figure 1:**
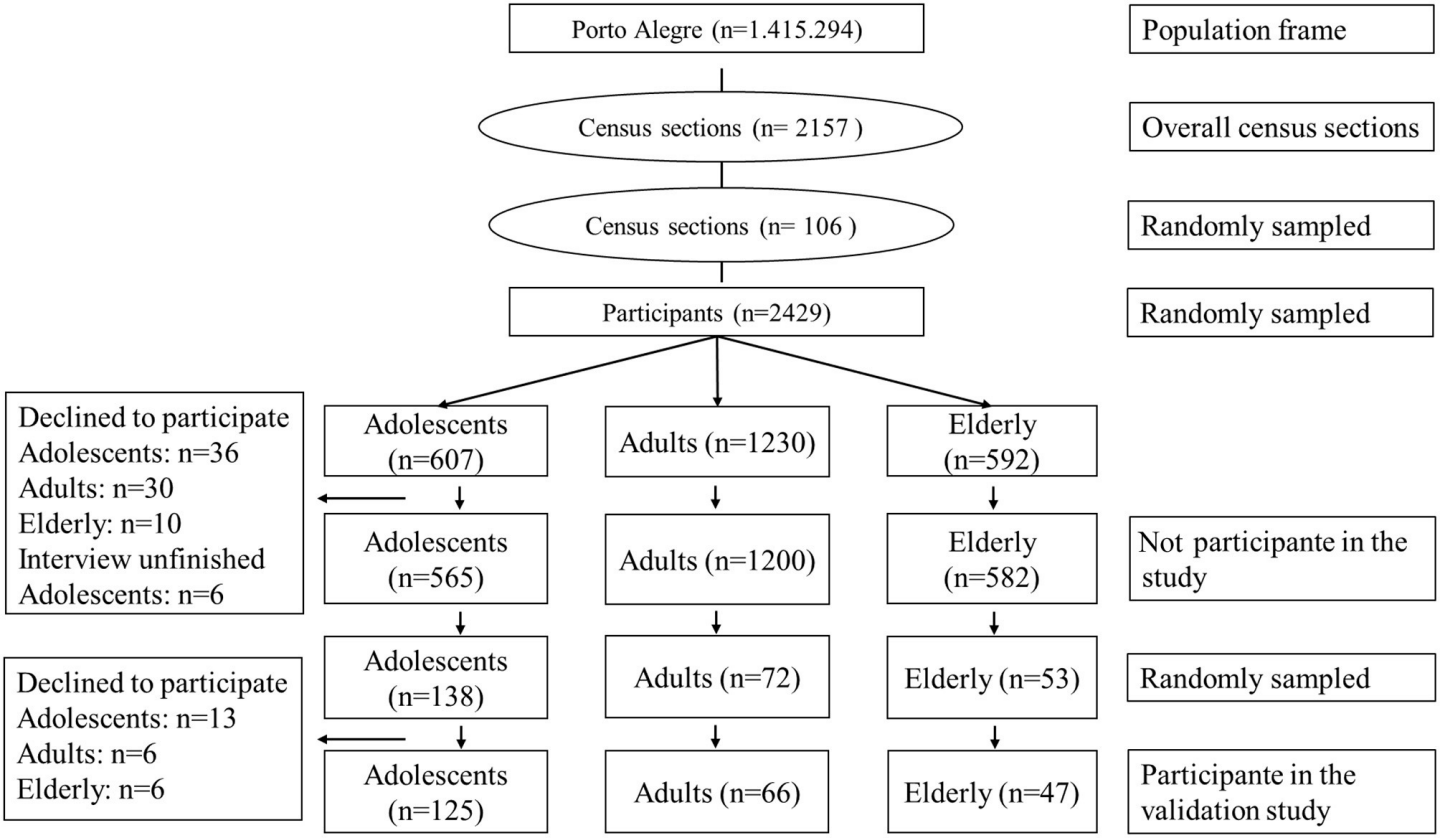
Flowchart describing the selection of participants.

The Syndrome of Obesity and Risk Factors for Cardiovascular Diseases (SOFT) study was a cross-sectional study conducted in Porto Alegre, southern Brazil, including a population-based sample of 2,157 adults, elderly, and adolescents selected using multistage sampling. The SOFT study population enrolled in the SOFT-Food Frequency Questionnaire validation study is described in Figure 1. In-person interviews were conducted at the study participant's household. Further details of the study are described elsewhere (21, 22). To validate the Food Frequency Questionnaire, participants responded to a 48-h dietary recall survey used as the reference method. The evaluation of adolescents, adults, and elderly participants were performed in public schools, universities, and recreational areas visited regularly by the elderly population, respectively, in Porto Alegre and surrounding areas. We used information on energy and nutrients intake collected from the 237 participants (108 men and 129 women, from 11 to 90 years old). The sample size was defined as the number of participants necessary to validate the Food Frequency Questionnaire as described elsewhere (22). Participants provided written consent. The Committee of Ethics in Research of Hospital de Clínicas de Porto Alegre approved the study.

### Diet Assessment

Usual food and nutrient intake were collected using 48-h dietary recall by trained interviewers (nutritionists), who asked participants about diet, particularly preparation methods, ingredients of hand-made recipes, quantities, portion sizes, and types and brands of industrialized food products. Participants were interviewed in their homes by nutritionists and graduate students following a standard protocol. The intake of food items was registered, including details of preparation methods, hand-made recipes, quantities and the size of portions, and the brands of industrialized products. Participants reported the size of portions and kitchen utensils assisted by a photo album (23) of 60 photographs of kitchen utensils used for cooking or setting the table, with varying sizes and capacities. Quality control procedures included a high supervision rate during data collection and repeated interviews using the same instrument, with additional questioning if needed for clarification. The calculation of total energy intake per day was based on the Brazilian Food Composition Table, fourth edition (24), and the National Nutrient Database for Standard Reference from the United States Department of Agriculture (25). Because the data collection forms were hand-filled, serving sizes were converted into weight and volume using the table to assess food intake in serving sizes (26). Dietary intake was calculated as energy, in calories per day; protein, carbohydrate, total fat, and total fiber in grams per day; and cholesterol, calcium, magnesium, vitamin C, saturated fat, monounsaturated fat, and polyunsaturated fat in milligrams per day.

### Statistical Analysis

Participants were grouped per gender (male and female) and age (adolescents from 11 to 19 years old, adults from 20 to 59 years old, and elderly from 60 to 90 years old). Nutrient intake was adjusted for energy intake using the residual method (27).

To assess within- and between-person variation, we assessed the variability of energy and nutrients over 2 days of dietary intake. The intake of energy, macronutrients, cholesterol, total fiber, and micronutrients was transformed using the Box-Cox method using R statistical software (28). The within- and the between-person variances ($S_{w}^{2}$ and $S_{b}^{2}$) were estimated using analysis of variation. We calculated the sources of within- and between-person variation using the equations described in Table 1 (4, 12–17). Our cutoffs were based on other studies (13–17) and sensitivity analysis. Thus, the percentage of deviation from the individual average and the sample average was defined as an acceptable error of 10% and an expected correlation between the usual and the observed nutrient intake of 0.95. Other cutoffs were insufficiently sensitive to detect the within-person variation. Statistical analysis was performed using SPSS 18.0.

**Table 1:** Equations description.

***N***	**Description**	**Equation**
1	Coefficient of variance within-person	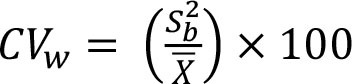
2	Coefficient of variance between-person	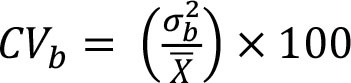
3	Variance ratio	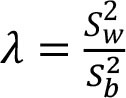
4	Percentage of deviation from the population average for the individual (Di)	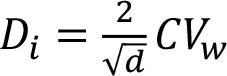
5	Percentage of deviation from the population average for the group (D_0_)	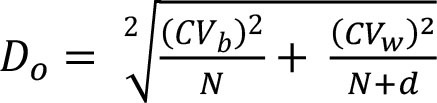
6	Number of days required to assess the usual energy and nutrient intake considering an acceptable error	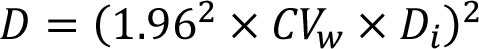
7	Sample size, where *D_0_* was the percentage of deviation (error of 10%)	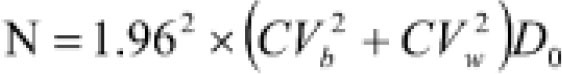
8	Correlation between the observed and the usual intake	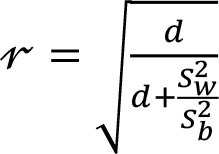
9	Number of days required to assess the usual energy and nutrient intake considering an expected correlation between the true and the estimated energy intake	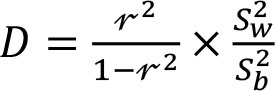

*here the (D_i_) was the percentage of deviation of each participant average of energy and nutrient intake, which is used to calculate how many days of diet assessment are necessary to estimate the usual energy and nutrient intake accurately. The (D_0_) was the percentage of deviation of each group (e.g., male, female, adolescents, adults, and elderly) from the population average of energy and nutrient intake, which was used to calculate the sample size of each group, considering a given number of days. The number of days necessary to estimate the usual energy and nutrient intake for the overall population and groups was assessed based on an acceptable percentage of deviation from the individual average (D_i_ = 10%), and the sample size was calculated using an acceptable percentage of deviation from the sample average (D_0_ = 10%) [16, 17] Where “d” is the number of days observed; is the average of energy and nutrients in this survey, and “N” is the number of overall population or the strata (e.g., group of gender, age). The “d” was represented by the 2 days of data collected using the 48-h DR. Both D_i_ and D_0_ were expected to be within a confidence of interval of 95%. The “D” was the number of days required to assess the usual intake of energy and nutrients and the Di was the expected percentage of deviation (error) from the individual average (10%). In the equation 8, the “N” represented the sample size, where D_0_ was the percentage of deviation (error of 10%). The number of days required to estimate the usual nutrients intake was also estimated using the correlation between the observed and the usual intake (r) by equation 9. The correlation was calculated using the equation 9, where r^2^ is the expected correlation between the usual and the observed nutrient intake (0, 95) (13, 14, 17)*.

## Results

There were 237 study participants, including 129 were women, 126 children 11–19 years old, and 46 were elderly (60–90 years old). Among the entire group of participants, the within-person coefficient of variation was lower than between-person variation for most nutrients, except for calcium (${\text{CV}}_{\text{w}}^{2}$ = 40.8 and ${\text{CV}}_{\text{b}}^{2}$ = 38.4%), magnesium (${\text{CV}}_{\text{w}}^{2}$ = 27.4 and ${\text{CV}}_{\text{b}}^{2}$ = 18.7%), and monounsaturated fat (${\text{CV}}_{\text{w}}^{2}$ = 20.0 and ${\text{CV}}_{\text{b}}^{2}$ = 17.3%). In women, there was a higher within-person variation than between-person variation for magnesium intake (${\text{CV}}_{\text{w}}^{2}$ = 28.3 and ${\text{CV}}_{\text{b}}^{2}$ = 91.8%) and in men, for vitamin C (${\text{CV}}_{\text{w}}^{2}$ = 83.7 and ${\text{CV}}_{\text{b}}^{2}$ = 36.1%) (Table 1). The magnesium intake for the entire group of participants had the highest variation ratio (λ = 2.14), followed by magnesium among women (λ = 2.11) and calcium in the entire population group (λ = 1.13). Men had a lower variation ratio in general, ranging from 0.005 for carbohydrates to 0.76 for calcium (Table 2). The correlation between the usual or true, and the estimated (as calculated based on this data) energy and nutrient intake was high for most nutrients; however, it was a little lower for magnesium (*r* = 0.69), calcium (*r* = 0.80), and iron (*r* = 0.84) intake in the entire group of participants, for magnesium (*r* = 0.70) and iron (*r* = 0.86) intake in women, and for calcium intake of men (*r* = 0.85) (Table 2).

**Table 2:** Coefficient of variation within person (CV_w_) and between person (CV_b_), variance ratio (λ), and correlation between observed and usual energy and nutrient intake (*r*^2^) for the whole sample and by gender.

**Nutrients**	**Whole sample**				**Women** ***N=*** **129 (54%)**				**Men** ***N=*** **108 (46%)**			
	**CV_**w**_**	**CV_**b**_**	**λ**	***r^2^***	**CV_**w**_**	**CV_**b**_**	**λ**	***r^2^***	**CV_**w**_**	**CV_**b**_**	**λ**	***r^2^***
Energy (kcal)	2.9	17.3	0.03	0.99	7.1	12.7	0.15	0.96	3.3	7.1	0.04	0.99
Protein (g)	9.2	22.2	0.17	0.96	14.3	47.9	0.37	0.92	1.6	7.1	0.01	1.00
Carbohydrate (g)	5.6	24.4	0.05	0.99	6.3	7.7	0.06	0.99	1.5	2.3	0.00	1.00
Total Fat (g)	2.9	26.1	0.01	1.00	12.1	30.7	0.22	0.95	8.8	22.6	0.12	0.97
Cholesterol (mg)	32.0	44.8	0.51	0.89	31.1	11.2	0.46	0.90	14.0	5.3	0.11	0.97
Total Fiber (g)	29.6	39.9	0.55	0.89	30.7	53.7	0.56	0.88	10.2	20.0	0.07	0.98
Calcium (mg)	40.8	38.4	1.13	0.80	25.7	11.1	0.42	0.91	32.1	15.0	0.76	0.85
Iron (mg)	40.6	48.7	0.69	0.86	49.0	70.7	0.86	0.84	7.5	15.1	0.03	0.99
Magnesium (mg)	27.4	18.7	2.14	0.69	28.3	91.8	2.11	0.70	9.8	37.1	0.30	0.93
Potassium (mg)	11.3	14.9	0.57	0.88	11.6	33.3	0.55	0.89	4.1	14.6	0.09	0.98
Vitamin C (mg)	52.8	707.5	0.40	0.91	0.5	0.2	0.00	1.00	83.7	36.1	1.10	0.80
Saturated Fat (mg)	23.3	43.9	0.34	0.93	18.1	32.8	0.19	0.95	14.8	27.1	0.14	0.97
Mono unsaturated fat (mg)	20.0	17.3	0.26	0.94	17.2	31.7	0.19	0.96	11.0	20.2	0.08	0.98
Polyunsaturated fat (mg)	18.8	22.2	0.18	0.96	16.0	20.4	0.12	0.97	10.3	16.8	0.06	0.98

Like for age (Table 3), within-person variance varied substantially for micronutrient intake. In general, the within-person variance was lower for participants 60–90 years old, except for vitamin C (CV_w_ = 71.6 in the elderly and CV_w_ = 19.3 in adults). In adolescents, the correlation between and observed intake was perfect for total fat and cholesterol. There was a perfect correlation between usual and observed protein intake, total fiber, calcium, potassium, saturated fat, and the elderly, for carbohydrate and total fiber in adults. Table 4 shows that the sample size to assess macronutrient intake in the whole population was smaller than for other nutrients. To estimate the energy and the total fat intake using a 48-h dietary recall to assess 2 days of dietary intake, 58 and 56 participants, respectively, would be necessary. More significant numbers of participants would be required to assess protein and carbohydrate intake (181 and 110 participants, respectively). The sample size differed substantially for each nutrient and participant group. To assess the intake of vitamin C using a 48-h dietary recall to assess 2 days of dietary intake, 1,641 men would be necessary but only nine women. This is a result of the much lower vitamin C within-person variation for women than for men that resulted in a perfect correlation between usual and observed intake for women (*r* = 1.00) and a lower correlation for men (*r* = 0.80) (Table 2). A similar phenomenon was sought for cholesterol intake. For cholesterol, it would be necessary to consider 636 adults and 653 older adults (Table 4), but only 28 adolescents, because, using a 48-h dietary recall to assess 2 days of cholesterol intake, the correlation between usual and observed intake was lower for adults (*r* = 0.88) and the elderly (*r* = 0.88) but higher for adolescents (*r* = 1.00) (Table 2). This finding suggests that the elderly population varied more in terms of intake of food sources of cholesterol from day to day than did adolescents.

**Table 3:** Coefficient of variation within person (CV_w_) and between person (CV_b_), variance ratio (λ), and correlation between observed and usual energy and nutrients' intake (*r*^2^) by age.

**Nutrients**	**11–19 years old** ***N=*** **126 (53%)**				**20–59 years old** ***N=*** **65 (27%)**				**60–90 years old** ***N=*** **46 (19%)**			
	**CV_**w**_**	**CV_**b**_**	**λ**	***r^2^***	**CV_**w**_**	**CV_**b**_**	**λ**	***r^2^***	**CV_**w**_**	**CV_**b**_**	**λ**	***r^2^***
Energy (kcal)	5.2	16.0	0.11	0.97	8.2	18.2	0.20	0.95	7.7	15.1	0.26	0.94
Protein (g)	4.9	21.3	0.05	0.99	1.4	24.0	0.00	1.00	15.0	20.6	0.53	0.89
Carbohydrate (g)	13.9	21.8	0.40	0.91	12.1	24.6	0.24	0.94	2.3	22.4	0.01	1.00
Total Fat (g)	1.2	25.0	0.00	1.00	7.8	27.3	0.08	0.98	14.7	25.2	0.34	0.92
Cholesterol (mg)	1.4	46.0	0.00	1.00	32.4	42.4	0.59	0.88	33.3	44.5	0.56	0.88
Total Fiber (g)	35.7	37.5	0.90	0.83	5.8	44.0	0.02	1.00	1.0	39.1	0.00	1.00
Calcium (mg)	41.9	37.4	1.26	0.78	2.0	41.9	0.00	1.00	24.7	36.7	0.45	0.90
Iron (mg)	38.1	41.6	0.84	0.84	10.1	54.5	0.03	0.99	14.1	50.9	0.08	0.98
Magnesium (mg)	28.7	17.7	2.62	0.66	3.6	21.3	0.03	0.99	19.0	17.8	1.13	0.80
Potassium (mg)	14.6	15.2	0.93	0.83	2.0	15.9	0.02	1.00	4.0	12.8	0.10	0.98
Vitamin C (mg)	39.7	449.2	0.22	0.95	19.3	3767.9	0.06	0.99	71.6	1408.6	0.73	0.86
Saturated Fat (mg)	28.1	46.9	0.51	0.89	4.5	40.9	0.01	1.00	11.3	38.2	0.08	0.98
Mono unsaturated fat (mg)	25.7	16.0	0.44	0.90	12.7	18.2	0.10	0.98	19.1	15.1	0.27	0.94
Polyunsaturated fat (mg)	8.1	21.3	0.03	0.99	12.4	24.0	0.09	0.98	47.2	20.6	1.53	0.75

**Table 4:** Sample size and number of days needed to estimate the energy and nutrients' intake using a 48-h dietary recall for the whole population and by gender and age.

**Nutrients**	**Whole sample**			**Men**			**Women**			**11–19 years old**			**20–59 years old**			**60–90 years old**		
	**SS**	**Nd^**1**^**	**Nd^**2**^**	**SS**	**Nd^**1**^**	**Nd^**2**^**	**SS**	**Nd^**1**^**	**Nd^**2**^**	**SS**	**Nd^**1**^**	**Nd^**2**^**	**SS**	**Nd^**1**^**	**Nd^**2**^**	**SS**	**Nd^**1**^**	**Nd^**2**^**
Energy (kcal)	58	7	1	64	9	1	139	36	2	102	23	2	160	47	3	150	65	3
Protein (g)	181	151	3	32	6	1	281	308	4	96	47	1	27	3	1	294	552	6
Carbohydrate (g)	110	18	1	30	1	1	123	21	2	272	121	5	237	84	3	44	3	1
Total Fat (g)	56	8	1	171	81	2	243	153	3	24	2	1	153	56	2	288	262	4
Cholesterol (mg)	626	407	6	275	78	2	609	385	5	28	1	1	636	423	6	653	444	6
Total Fiber (g)	581	637	6	200	80	2	602	652	6	699	1,000	9	115	22	1	20	1	1
Calcium (mg)	799	673	11	629	420	8	503	266	5	822	716	13	39	2	1	485	250	5
Iron (mg)	796	1,089	7	147	44	1	961	1,426	9	747	1,184	9	198	60	1	276	141	2
Magnesium (mg)	538	1,199	21	193	175	4	554	1,150	21	562	1,568	25	70	14	1	372	656	11
Potassium (mg)	221	175	6	81	28	2	226	162	6	286	282	10	39	4	1	77	35	2
Vitamin C (mg)	1,035	1,110	5	1,641	2,828	11	9	1	1	778	628	3	378	149	2	1,402	2,025	8
Saturated Fat (mg)	457	390	4	289	162	2	355	234	3	550	581	6	88	14	1	222	97	2
Mono-unsaturated fat (mg)	392	288	3	216	87	2	336	214	3	504	483	5	248	105	2	374	335	4
Polyunsaturated fat (mg)	368	207	3	203	69	2	313	140	2	158	36	1	242	95	2	925	1,720	15

SS—Sample size for data collected with one 48-h dietary recall, accepting 10% of deviation of the average (Error). N = 1.96^2^(${\text{CV}}_{b}^{2}+$${\text{CV}}_{w}^{2}$) D_0_ by Piwoz et al. (29). Nd^1^–Number of days ^1^for data collected with one 48-h dietary recall, capturing a variation of 10% (accepted error) around individual average. D = (1.96 x CV_W_/D_i_)^2^ by Beaton (12). Nd^2^–Number of days ^2^for data collected with one 48-h dietary recall, expecting a correlation of 0.95 (confidence level) between estimated and usual intake of energy and nutrients. D = (r^2^/1-r) *(Sw^2^/SB^2^) by Nelson et al. (13).

Using one 48-h dietary recall to assess 2 days of dietary intake, capturing 10% of the within-person variation (the percentage of variation around individual averages) in the entire group of participants, 7, 8, and 18 days would be necessary to assess the usual intake of energy, total fat, and carbohydrate intake, respectively; however, a much larger number of days would be necessary to assess protein intake (151 days) (Table 4). The within-person variation had the most significant impact on the number of days necessary to assess usual dietary intake. The larger the within-person variance and the variance ratio (Tables 2, 3), the greater the number of days needed to assess usual nutrient intake. For example, in the entire group of participants, the number of days to assess the usual intake of magnesium (1,119 days), vitamin C (1,110 days), and iron (1,089 days) (Table 4) accepting an error of 10% around the individual average were similar. In these examples, the numbers of days necessary to assess usual dietary intake was more dependent on the within-person variance ratio (λ) than the between-person variance (magnesium CV_w_ = 27.4 and λ = 2.14; vitamin C CV_w_ = 52.8 and λ = 0.40; iron CV_w_ = 40.6 and λ = 0.69) (Table 2).

Adolescents needed 24, 54, 422, and 59 fewer days than adults to assess the intake of energy, total fat, cholesterol, and polyunsaturated fat, respectively. They required 42, 260, 443, and 1,684 fewer days than the elderly to assess the same nutrients (Table 4). The numbers of days necessary to assess carbohydrates and total fiber were higher among adolescents than adults (37 and 118 days, respectively) and the elderly (978 and 999 days, respectively). Overall, the adult population needed fewer days than adolescents and the elderly to assess intake of protein (44 and 549 days less, respectively than adolescents and elderly), calcium (714 and 248 days less, respectively), iron (1,124 and 81 days less, respectively), magnesium (1,554 and 642 days less, respectively), potassium (278 and 31 days less, respectively), vitamin C (479 and 1,876 days less, respectively), and saturated fat (567 and 83 days less, respectively).

## Discussion

We found that 48-h dietary recall provided ineffective measurement of within-person variation. This finding means that, for some nutrients, 48-h dietary recall informs as much as a single 24-h dietary recall, depending on age and gender, although the between-person variation was captured acceptably. Because the within-person variation was potentially underestimated, the sample size and number of days necessary to assess the usual intake of energy and nutrients are more likely to represent one instead of 2 days of dietary intake.

We found lower within-person variation than between-person variation for intake of energy, macronutrients, and micronutrients than other studies that used two or more non-consecutive (8, 30) and consecutive (31) 24-h dietary recalls for data collection. Oh and Hong, in an elderly population from Inchon, Korea, observed that within-person variation was twice as high in men and 1.5 as high in women than between-person variation (17). Compared to a study based on data collected using two 24-h dietary recalls in adolescents, the within-person variation of energy was 280-fold, and the variance ratio was 20 times higher than what we observed in the present study (30).

Several conditions may influence the variation ratio. We used an unadjusted statistical model that could have influenced the results. For example, in the analysis of data collected using 24-h dietary recall, the energy variation ratio of men was 1.07 in the analysis adjusted for the fixed effect of gender, age, education, smoking, size of family, and season; the variation was 0.49 in the unadjusted analysis. In the same study, the variation ratio was lower in the unadjusted models for women (16). Macronutrient intake influences energy balance, remaining constant throughout the days (1); therefore, the intake of each nutrient is expected to vary more than energy, in general (16, 30). Studies based on the 24-h dietary recall have shown that intake of protein, total fat, and carbohydrate is naturally higher than the energy intake in general (16, 32); however, there may be lower for women's intake of protein (17) and carbohydrates (30, 32). In the present study, cholesterol, fiber, and micronutrients had a higher variation ratio than energy and macronutrients, as observed in previous studies, although those studies were based on the 24-h dietary recall (8, 13, 16).

Consequently, we acknowledge that the 48-h dietary recall could capture lower energy and nutrient variability than two 24-h dietary recalls. In addition, 48-h dietary recall and the food record, both based on recall, performed better in men with significant differences for nutrient intakes when comparing the former to the food record. This finding suggests that males had less variation in their intake over the 48-h dietary recall than subsequent (5) days recorded in the food record (1). In this and other studies, the sample size and the number of days required to assess the energy and nutrient intake, accepting an error of 10% around the individual average, or based a correlation of 0.95 between the and the estimated energy and nutrients intake, differed according to gender and age group and ranged widely among nutrients (1, 8, 12, 17, 30).

Using the 48-h dietary recall method to collect the data was as much accurate as one 24-h dietary recall to assess the intake of vitamin C for women in contrast to seven nutrients (protein, total fat, cholesterol, total fiber, calcium, iron, magnesium, and potassium) for adult men and women. Two days of nutrient intake showed a perfect correlation between usual and estimated nutrient intake for a few nutrients, and this result varied with the population group. These perfect correlations suggest that one 48-h dietary recall and two consecutive 24-h dietary recalls generate the same results. Studies using non-consecutive 24-h dietary recall or food records showed lower correlations between the usual and the estimated nutrients (8, 17, 30).

### Pros & Cons of Sample Size vs. Repeated Measures

Study designs demand a sampling plan to ensure minimal cost and maximum accuracy (12). From this point of view, using 24-h dietary recall is preferable because the 48-h dietary recall did not provide extra value but rather demanded additional effort from the participant to record in detail the food intake of 2 days instead of one (33). If the within-person variation is not essential, a 48-h dietary recall could be helpful because it appears to capture the between-person variation appropriately. The result of this study might be applicable to plan sample size calculations and the number of days for dietary assessment, considering that the 48-h dietary recall poorly captures the energy and nutrient day-to-day variation. The latter observation certainly affects the calculation of the sample size and number of days required.

### Strengthens and Limitations

This study demonstrated that dietary assessment using the 48-h dietary recall has limitations for some nutrients; however, it can be helpful for others, depending on the population. The strength of this study is that it is the first analysis of sources of variations of nutrient intake of data using 48-h dietary recall. This is particularly important because our findings support further studies designed to compare the 48-h dietary recall methodology with other reliable methods, including biomarkers, the 24-h dietary recall, and daily diet records. However, this study has limitations that must be circumvented in the future. Because the study participants were selected randomly in a population-based setting, our findings might be appropriate for adolescents and adults living in urban areas of southern Brazil but not for rural areas. Furthermore, for a more accurate comparison between the energy and nutrient variation observed for one 48-h dietary recall compared to two consecutive 24-h dietary recalls, information from both methods should be collected in the same population and during the same time frame.

## Conclusions

Energy and macronutrient variability tend to be lower than those of fiber, cholesterol, and micronutrients; the overall variability is strongly influenced by gender and age group and can be captured using either the 24-h dietary recall or the 48-h dietary recall. The 48-h dietary recall might be helpful to assess energy and nutrients because it captures between-person variation. For example, it can be used to assess energy, protein, total fat, and polyunsaturated fat in the overall sample; however, among the elderly, it might introduce higher variation than in other population groups for the assessment of energy, protein, total fat, vitamin C, and polyunsaturated fat. Nevertheless, within-person variation is considerably lower than between-person variation when the data are collected using the 48-h dietary recall, and this finding contradicts the assumption that one 48-h dietary recall could capture the variability of two 24-h dietary recalls accurately. Our study did not intend to recommend how many days are necessary to assess dietary intake using the 48-h dietary recall to other populations; instead, its purpose was to highlight the limitations and strengths of this methodology, showing potential influences of the method and population characteristics on usual nutrient intake estimations, to support decision-making and research to assess the validity of this methodology.

## Data Availability Statement

The original contributions presented in the study are included in the article, further inquiries can be directed to the corresponding author/s.

## Ethics Statement

The studies involving human participants were reviewed and approved by Committee of Ethics in Research of Hospital de Clinicas de Porto Alegre, and approved under the register number GPPG-00176.

## Author Contributions

SR planned and performed the statistical analysis and wrote the manuscript. SF conceived and designed the data collection, took part in the analysis, and revised the manuscript. All authors contributed substantially to the work and approved the final version of the manuscript.

## Conflict of Interest

The authors declare that the research was conducted in the absence of any commercial or financial relationships that could be construed as a potential conflict of interest.
